# Ultrasound image attributes of human ovarian dominant follicles during natural and oral contraceptive cycles

**DOI:** 10.1186/1477-7827-3-12

**Published:** 2005-04-13

**Authors:** Rebecca L Birtch, Angela R Baerwald, Olufemi A Olatunbosun, Roger A Pierson

**Affiliations:** 1 Department of Obstetrics, Gynecology and Reproductive Sciences, College of Medicine, University of Saskatchewan, Saskatoon, Saskatchewan, Canada

## Abstract

**Background:**

Computer-assisted analyses were used to examine ultrasound image attributes of human dominant ovarian follicles that developed during natural and oral contraceptive (OC) cycles. We hypothesized that image attributes of natural cycle follicles would quantitatively differ from those in OC cycles and that OC cycle follicles would possess image attributes indicative of atresia.

**Methods:**

Dominant ovarian follicles of 18 clinically normal women were compared using transvaginal ultrasonography for the 7 days before ovulation during a natural cycle (n = 9) or the 7 days before peak estradiol in women using OC (n = 11). Follicles were analyzed using region and line techniques designed to compare the image attributes numerical pixel value (NPV), pixel heterogeneity (PH) and area under the curve (AUC).

**Results:**

NPV was higher in OC cycle follicles with region analysis and tended to be higher with line analysis (p = 0.005 and p = 0.06, respectively). No differences were observed in two other image attributes (AUC and PH), measured with either technique, between natural and OC cycle follicles.

**Conclusion:**

The increased NPV value of OC cycle follicles and lack of differences in PH and AUC values between natural cycle and OC cycle follicles did not support the hypothesis that OC cycle follicles would show ultrasonographically detectable signs of atresia. Image attributes observed in OC cycle follicles were not clearly indicative of atresia nor were they large enough to preclude preovulatory physiologic status in OC cycle follicles.

## Background

Diagnostic gray-scale ultrasonography has revolutionized the study of ovarian biology in animals and humans because it allows researchers and clinicians to assess the development of individual follicles in a direct, non-invasive, and atraumatic manner without interruption or distortion of ovarian function. Prior to the introduction of ultrasonography, histological slices of ovarian tissue were used to elucidate ovarian follicular development; however, histologic investigation only provides information about a single time point and does not permit assessment of follicular function over time. Further, histology cannot be used for time-series studies in humans.

Animal models have been developed to elucidate the basic mechanisms of ovarian function in humans and to overcome ethical impossibilities of some aspects of research in humans. To date, however, no appropriate animal models are available to elucidate the physiological effect of oral contraceptives (OC) on human ovarian function due to species specific differences in the metabolism of the exogenous estrogen and progestins used to control reproductive function. Nor do non-invasive techniques exist that will allow for the determination of the physiological status of individual ovarian follicles with a single observation. However, new technologies involving computer assisted image analysis to elucidate a follicle's physiologic status show promise [1,2].

Quantitative changes in ultrasound image echotexture as indicators of physiological function of ovarian structures have been described in domestic animal models [3-8]. The validity of the image analysis technique has been verified through correlation of ultrasound image attributes with histologic attributes [3,4,8]. Similar studies in humans are ethically impossible; however, information generated in animal studies may be applied to human imaging based studies [3-6,9,10]. The overall goal of this line of research in our laboratory is to elucidate physiologic status of dominant follicles with non-invasive ultrasonography in humans. Imaging-based techniques that could be used to determine follicular health would obviate the ethical and logistical limitations associated with ovarian function research in women.

It has recently been discovered that women grow follicles in two or three follicular waves during each natural menstrual cycle [11,12]. The pattern of folliculogenesis is similar in women to those observed in several species of domestic animals (bovine, equine, caprine and ovine) [9,13-17]. A follicular wave is defined as a cohort of follicles that enter the antral growth phase synchronously. Growth of all follicles in the cohort continues until one follicle is physiologically selected as the dominant follicle. The dominant follicle continues its development to pre-ovulatory diameter while the remaining follicles in the cohort undergo atresia. The dominant follicle will ovulate if the appropriate hormone signals (i.e., mid-cycle luteinizing hormone surge) are provided. If the hormone signals which trigger ovulation are not provided, the dominant follicle enters a static phase and remains approximately the same diameter until it enters the regressing phase, when it decreases in diameter until it is no longer detectable.

Dominant follicles develop in women during compliant use of OC, with most dominant follicles initiating growth during the hormone-free interval [18-22]. Ovulatory follicles that develop during spontaneous natural cycles are presumed to be healthy because they typically ovulate. It is not known whether dominant follicles of ostensibly preovulatory diameter that arise during OC use have the same physiologic status and/or ovulatory capacity as natural preovulatory follicles.

The primary objective of the present study was to assess ultrasonographic image attributes of dominant ovarian follicles during the final stages of development in natural menstrual cycles and OC cycles. We hypothesized that image attributes of follicles from natural cycles would quantitatively differ from OC cycle follicles due to the effects of the exogenous hormones on OC cycle follicles and that the image attributes of OC cycle follicles would be consistent with those indicative of atresia.

## Materials and Methods

This study was a retrospective, observational study designed to evaluate and compare ultrasound image attributes of dominant ovarian follicles (≥ 10 mm in diameter) during natural and OC cycles. Images of twenty (n = 20) dominant follicles were analyzed. The images were obtained in two previous studies designed to characterize ovarian follicular wave dynamics during spontaneous natural cycles [11,12] and OC cycles [22]. The inclusion and exclusion criteria of both studies were similar. Participants were assessed, by history and physical examination, to be healthy women of reproductive age (28.0 ± 0.14 years, natural cycles; 24.5 ± 0.02 years, OC cycles; mean ± SEM). All women in both studies had a history of normal menstrual cycles and could not have used OC for a minimum of three months before participating. In the OC trial, one of three different OC formulations were administered to thirty six women for three consecutive 28-day cycles; (i) 20 μg ethinyl estradiol (21)/100 μg levnogestrel (21) (n = 7), (ii) 30 μg ethinyl estradiol (21)/150 μg desogetrel (21) (n = 2), or (iii) 35 μg ethinyl estradiol (21)/180 μg norgestimate (7)/215 μg norgestimate (7)/250 μg norgestimate (7) (n = 2). Nine women grew 11 dominant follicles during compliant OC use. Two women grew dominant follicles during two separate cycles of OC-use; therefore, images from eleven dominant, ostensibly, ovulatory follicles were analyzed in the present study [22]. Images of nine dominant follicles from nine volunteers were randomly selected from natural cycle data to act as controls [11,12]. Blood samples were drawn every third day for women in the natural cycle trial and every second day once a follicle reached ≥ 14 mm diameter for women in the OC cycle trial.

A high resolution ultrasound instrument equipped with 5–9 MHz multi-frequency intravaginal convex array transducer (ATL Ultramark HDI 5000, Advanced Technologies Laboratories, Bothwell, WA, USA) was used. Settings of the ultrasound instrument that affect image attributes (beam focus, overall time-gain, near-field and far-field gain) were standardized to the same predetermined standards for both studies. Each image was digitally acquired and transferred into a customized computer database during the ultrasound examinations. Images of the dominant follicle acquired in largest cross-sectional diameter with the fewest image artifacts were selected for image analysis. All image analyses were performed by the same individual (RLB). Blinding was not possible because each image contained identifying study information.

Image attributes of dominant follicles were analyzed using a graphics workstation equipped with a customized software (SYNERGYNE 2©, Saskatoon, SK, Canada) integrating complex algorithms designed for ultrasonographic image analysis [1,2]. Each image was analyzed with two different techniques: i) region analysis and ii) line analysis designed to quantify gray-scale values of selected regions [1,2]. Three image attributes were quantified: i) numerical pixel value (NPV) defined as the mean pixel gray-scale value of the sampled pixels, ii) pixel heterogeneity (PH) defined as the standard deviation of the mean gray-scale values of the sampled pixels and, iii) area under the curve (AUC) defined as the area of the sampled region in pixels.

Briefly, region analyses involve overlaying a computer-generated grid onto a selected area of the 2-dimensional image of a follicle to generate a 3-dimensional framework representing processed pixel intensities. Placing a computer-generated opaque "film" or blanket over the wire framework representing pixel values comprising the ultrasound image yields a 3-dimensional contoured surface. This technique allows discriminatory examination of the surfaces and rapid visual assessment of ultrasonographic attributes associated with follicle health (state of viability or atresia); [2]. Region analyses were used to measure NPV, PH and AUC within a region of the follicle wall. A one-pixel-wide line was used to outline and isolate the largest continuous portion of follicular wall extending from the peripheral antrum to the stroma with the fewest image artifacts. The antrum-wall interface was defined as the last pixel along the line in which a sequential rise in gray-scale occurred [4].

Line analysis places a line across a specified section of the image of the follicle to produce a graph of the pixel intensities along the line displayed. The graph depicts the amplitude of the echoes located along the line [2]. Line analysis was used to measure NPV, PH and AUC of a single line across the follicle wall from the antrum-wall interface to the ovarian stroma using two straight three-pixel wide lines. The two linear measurements were taken between the 4 and 9 o'clock positions of the follicle image.

Ovulation was defined as the disappearance of a follicle greater than 15 mm in diameter identified the previous day and confirmed with visualization of a corpus luteum [11,12,23,24]. Ovulation was used as a reference point (Day 0) to standardize the data for natural cycle follicles. No follicles ovulated during OC cycles; therefore, OC cycle follicles were standardized to the day of peak estradiol concentration (Day 0) which was also defined as the last day of the growth phase. The day of peak estradiol concentration for each individual was determined by reviewing the estradiol profiles for each woman in the OC trial [22].

Ultrasound images were available for each of the seven days leading to ovulation for women in the natural cycle study and approximately every second day for seven days leading to peak estradiol levels for women participating in the OC trial. Data were standardized by aligning data from Day 0 as defined for each group. Image attributes on each of the seven days (Days -1 to -7) leading to ovulation during the natural cycle were compared to the corresponding days (Day -1 to -7) leading to peak estradiol concentrations during the OC cycle. Data were inadequate for statistical comparison of image attributes among the three different OC formulations, therefore data were combined.

Image attributes were compared using repeated measures analysis of variance (PROC MIXED, SAS/STAT, *v*8) for main effects of time and follicle type. Significance was set at p < 0.05. Results are expressed as the mean ± SEM.

## Results

**Figure 1:**
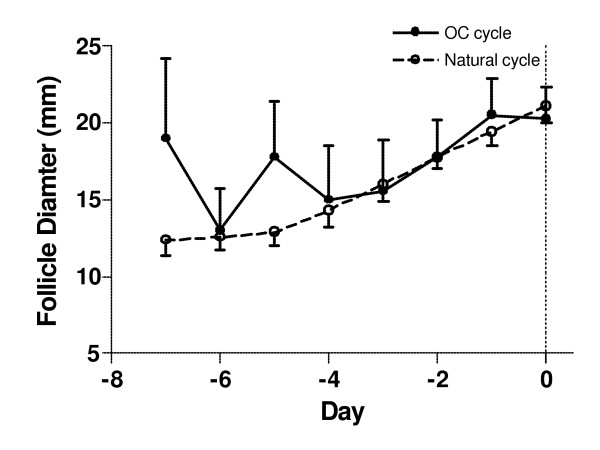
Growth profiles of natural and OC cycle follicles

**Figure 2:**
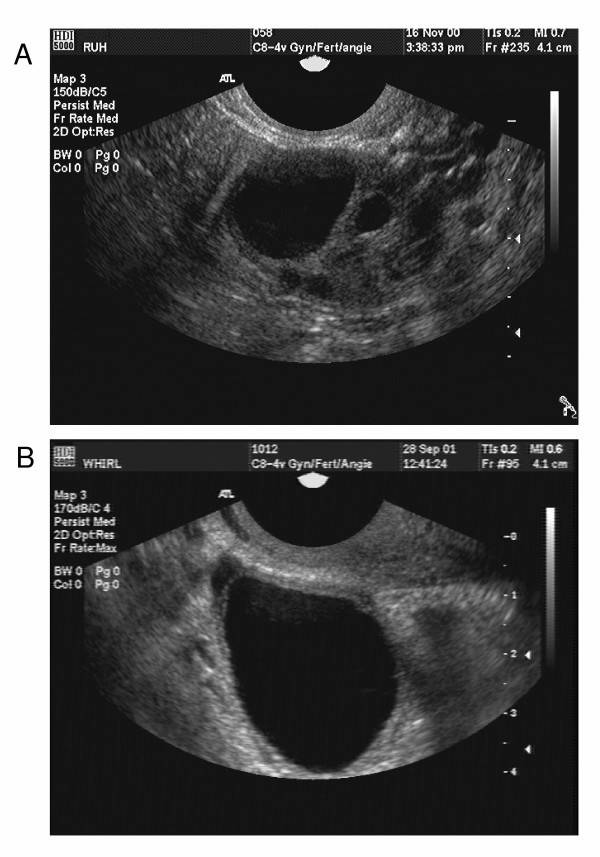
**Ultrasonographic images of a natural cycle and OC cycle follicles**. (a) Natural cycle follicle on Day 0, (b) OC cycle follicle on Day 0.

Peak estradiol-17β concentrations for OC cycle follicles were 170.1 ± 30.4 pg/mL (range, 35.1 – 364.5 pg/mL) [22]. Mean follicle diameter of OC cycle follicles at peak estradiol concentrations was 20.3 ± 2.0 mm. Mean estradiol concentrations for natural cycle follicles on Day -1 were 190.8 ± 19.6 pg/mL (range 20.0 – 340 pg/mL) [11]. Mean follicle diameter of natural cycle follicles on Day -1 was 21.1 ± 1.1 mm. Mean growth profiles for natural and OC cycle follicles are displayed in Figure 1. Ultrasonographic images of a natural cycle follicle one day before ovulation and an OC cycle follicle on the day of peak estradiol are shown (Figure 2,a and 2b, respectively).

**Figure 3:**
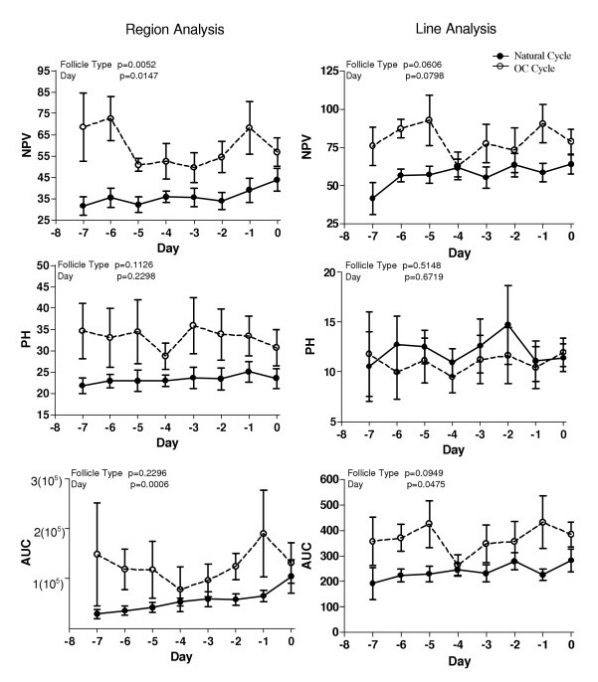
**Graphical representation of NPV, PH and AUC**. Mean ± SEM (a, b) numerical pixel value (c,d) pixel heterogeneity and (e, f) area under the curve obtained by region analysis (a, c, e) and line analysis (b, d, f) of natural cycle and OC-cycle follicles.

Numerical pixel values were higher (p = 0.005) for OC cycle follicles versus natural cycle follicles using region analyses and tended to be higher (p = 0.06) using line analysis for the 7 days prior to Day 0. Oral contraceptive cycle follicles had visually similar NPV for both region and line analyses; the values were initially high and then decreased to a nadir at Day -3 (region analysis) or Day -4 (line analysis) after which they increased until Day -1 and subsequently decreased on Day 0 (Figure 3a,b). Numerical pixel values for natural cycle follicles progressively increased from Day -7 (Region analysis; 31.64 ± 4.26, Line analysis; 41.66 ± 10.49) to Day 0 using line analysis (64.03 ± 6.37; p = 0.01) and tended to increase (43.80 ± 5.30; p = 0.08) with region analysis (Figure 3a,b).

Pixel heterogeneity values were not different between natural and OC cycle follicles for either region or line analyses (p = 0.113 and p = 0.515, respectively). No changes were observed over the seven days prior to Day 0.

Area under the curve evaluated using line analyses tended to be higher (p < 0.09) in OC cycle follicles; however, no differences were observed (p = 0.23) in follicle type using region analyses. Values for AUC for both natural and OC cycles increased as the interval to Day 0 decreased.

Nadirs in image attributes for the values representing AUC and PH were observed on Day -4 (Figure 3c–f).

## Discussion

The study of ovarian follicular dynamics in women taking hormonal contraceptives is a relatively new research area with profound clinical relevance. Follicular development in up to 50% of new OC cycles and up to a 25% ovulation rate have been reported [18]. Most follicular development observed in OC cycles is initiated during the hormone free interval of the OC cycle [22]. Our objective was to assess the indicators of follicular physiological status in women by comparing ultrasound image attributes of human dominant ovarian follicles leading to ovulation in natural cycles or peak estradiol concentrations in OC cycles. Numerical pixel values representing the relative brightness of the elements comprising the ultrasound image were the only image attributes that differed between natural and OC cycle follicles. Morphologic changes indicative of atresia in animal models are typically represented by high values of NPV [4,6]. These morphologic changes include, but are not limited to, an increase in the number of multivesicular bodies, lipid droplets and vacuoles within granulosa cells, dilation of the smooth endoplasmic reticulum and golgi apparatus and thecal cell hypertrophy/degeneration; which, when taken together, appear to affect the tissue properties enough to change the image attributes [25,26]. Although NPV were greater in OC cycle follicles, the increased values were not high enough to indicate poor physiologic status as described in studies using animal models [4,6]. The hypotheses that image attributes of natural and OC cycle would quantitatively differ and that OC cycle follicles would possess image attributes characteristic of atresia were only partially supported.

An interesting common observation in all image attributes of OC follicles that we evaluated fell to a nadir at Day -4, suggesting that an important physiological event occurs approximately four days before peak estradiol concentrations were attained. The image attributes of the values reflected in NPV and AUC in both natural and OC cycle follicles tended to increase from Day -4 to Day 0. This observation may be interpreted to mean that the follicles in both natural and OC cycles have similar physiologic status and undergo similar changes during the final stages of development. Classically described, ovulatory changes include thickening of the follicle wall, development of lipid inclusion granulosa and theca cells, hypertrophy of theca cells and increased vascularization of the theca layer [27,28]. Taken together, these developmental changes may be reflected as increases in AUC as the follicle wall thickens and increases in NPV as the follicle wall develops to fulfill its biological functions. Further research is needed to determine the physiologic changes responsible for changes in image attributes.

No OC cycle follicles analyzed in the present study ovulated. However, we suggest that follicles which develop during compliant OC use may differ in ovulatory potential because ovulation has been documented in up to 50% of follicles which develop to ostensibly ovulatory diameter during compliant OC use [18]. Differences in OC formulations (i.e. estradial concentrations and progestin type and associated activity) may have different physiological effects on follicular development and subsequent ovulatory ability, and individual responses to the different estrogen levels and the various progestins used in OC formulations may affect ovulatory ability differently. We were unable to test this hypothesis directly in the present study. Variations in image attributes of OC cycle follicles may be due to differences in physiologic status and ovulatory ability. Further research to determine the physiological mechanisms responsible for differences in ovulatory ability of OC cycle follicles needs to be completed.

Studies in domestic animals have demonstrated that ultrasound image attributes of ovarian structures are related to their physiologic function [3,4,8]. The present study provides rationale for exploring the hypothesis that the same is true for humans. In order to have a complete understanding of the relationship between image attributes and follicular development in humans, we must assess all stages of follicular development throughout the menstrual cycle, prediction of dysfunctional follicular development (i.e., hemorrhagic anovulatory follicles) and the effects of different OC formulations on follicle development. Once the association between image attributes and various conditions of follicular development are determined, follicular status could be assessed with a single ultrasound examination, obviating many ethical constraints that currently prevent progress in human ovarian follicular research.

Ovarian follicles provide a unique endocrine environment fundamental to normal oocyte development and competence. Abnormal follicular development can lead to incompetent oocytes; therefore it follows that image attributes of the follicle wall may be related to oocyte competence. Currently, image attributes only provide us with information about the physiologic status of the follicular wall. Image analyses do not provide information about the health and viability of the oocyte; however, this area is under active investigation [7].

The primary objective of this research was to evaluate and compare ultrasound image attributes of dominant follicles that develop during natural cycles and OC cycles. We were not able to examine the effect of different OC formulations on image attributes due to constraints imposed by study design. It is hoped that future research will explore the effects of different OC formulations on the image attributes of dominant follicles. Research to evaluate physiologic status may well hinge upon follicular response to administration of ovulation inducing doses of recombinant human chorionic gonadotrophin (hCG) in women who develop dominant follicles during OC cycles in concert with development of an animal model to test the biological effects of varying doses of ethinyl estradiol or different progestins on follicle and oocyte status.

## Conclusion

In conclusion, similarities in image attributes between natural and OC cycle follicles provides preliminary evidence that ultrasound image attributes of human follicles are associated with physiologic status during the growth phase and that follicles which develop during OC cycles have image attributes similar to those of natural cycle follicles. Only one of the three image attributes studied differed between natural and OC cycle follicles. The changes observed in OC cycle follicles were not clearly indicative of atresia nor were they large enough to preclude preovulatory physiologic status in OC cycle follicles.
